# Comprehensibility of a personalized medication overview compared to usual-care prescription drug labels

**DOI:** 10.3389/fphar.2022.1004830

**Published:** 2022-10-28

**Authors:** Laura Schackmann, Liset van Dijk, Anne E. M. Brabers, Sandra Zwier, Ellen S. Koster, Marcia Vervloet

**Affiliations:** ^1^ Nivel, Netherlands Institute for Health Services Research, Utrecht, Netherlands; ^2^ Groningen University Department of PharmacoTherapy, Epidemiology and Economics (PTEE), Groningen Research Institute of Pharmacy, Faculty of Science and Engineering, Groningen, Netherlands; ^3^ Faculty of Social and Behavioural Sciences, University of Amsterdam/Amsterdam School of Communication Research, Amsterdam, Netherlands; ^4^ Division of Pharmacoepidemiology and Clinical Pharmacology, Utrecht Institute of Pharmaceutical Sciences (UIPS), Faculty of Science, Utrecht University, Utrecht, Netherlands

**Keywords:** comprehensibility, (usual-care) prescription drug labels, medication overview, patient-tailored medication information, treatment adherence

## Abstract

Poor understanding of prescription drug label (PDL) instructions can lead to medication errors, suboptimal treatment (side) effects, and non-adherence. A personalized medication hard-copy overview listing PDL instructions and visual information may support patients in their medication use. This study aimed to investigate the comprehensibility of PDL instructions on a personalized medication overview compared to usual-care PDL instructions presented on a medication box. A hypothetical-online-experiment was set up, comparing groups of respondents exposed vs not exposed to the medication overview and who received PDL instructions for three, five, or eight medications. Participants were divided randomly in six groups. Online questionnaires were sent to a stratified sample of 900 members from the Nivel Dutch Healthcare Consumer Panel. Outcome measures included comprehension of instructions for medication use, e.g. how often, dose timing, usage advice and warnings for a medication with simple use instructions (omeprazol) and more complex use instructions (levodopa/carbidopa (L/C)). To analyze differences between experimental conditions ANOVA testing was used. 604 respondents (net response 67%) completed the questionnaires. Respondents exposed (E) to the overview gave a higher proportion of correct answers compared to non-exposed (NE) respondents for usage advice (L/C: mean 0.83, SD 0.4 E; 0.03, SD 0.2 NE, *p* < 0.001; omeprazol: mean 0.85, SD 0.4 E; 0.10, SD 0.3 NE, *p* < 0.001). Both groups gave the same proportion of correct answers (mean 0.80, SD 0.4, *p* = 1.0) for dose timing of omeprazol. More NE respondents gave correct answers for how often (mean 0.85, SD 0.4 NE; mean 0.76, SD 0.4 E, *p* = 0.02) and dose timing (mean 0.92, SD 0.3 NE; mean 0.86, SD 0.4 E, *p* = 0.04) of L/C. No differences were found regarding number of medications nor were interaction effects found between the number of medications and information type. As a medication overview contains additional information, it can be a good addition in supporting patients in their medication use compared to usual-care PDLs. Future research should focus on identifying patient groups who might benefit more from a medication overview, by testing the effect of such overview on this group.

## Introduction

Poor understanding of prescription drug label (PDL) instructions can lead to medication errors, side effects, suboptimal treatment effects and non-adherence (Davis et al., 2009; Bailey et al., 2015). PDLs are often the most read source of information before a patient starts using the medication (Webb et al., 2008), and they contain dosing instructions, usage recommendations and warnings (Wolf et al., 2007). On the PDL, there is only limited space, making it difficult to provide additional information (Maghroudi et al., 2020). Consequently, the information on the PDLs is often not comprehensible, as up to 50% of the adult population show limited understanding of PDLs, precautions, and medication warnings (Davis et al., 2006a; Davis et al., 2006b; Wolf et al., 2006; Wolf et al., 2007; Bailey et al., 2009; Wolf et al., 2010; Wolf et al., 2011; Bailey et al., 2012; Bailey et al., 2014).

Problems understanding medical information seem to be more common in certain patient groups, such as the elderly, people with limited health literacy, and people with language barriers (van Dijk et al., 2016). However, when it comes to PDLs also some with adequate health literacy skills find it difficult to understand and apply the usage instructions on PDLs. Previous research by Davis et al. (2006) showed that 37% of the interviewed patients, including those with adequate health literacy scores, did not understand instructions on the PDLs correctly (Davis et al., 2006a). To ensure understanding of instructions it is important to formulate instructions as clearly and explicitly as possible (Davis et al., 2006b; Bailey et al., 2012).

Researchers have long studied how to best provide comprehensive medication information related to medication use and understanding in a simplistic and practical manner. As such, numerous studies related to this topic have been published (Maghroudi et al., 2021). Studies have focused on factors such as, complexity of dosing instructions particularly in relation to patient health literacy (Beckman et al., 2005; Shrank et al., 2007; European Commission, 2009; Bailey et al., 2012; Emmerton et al., 2012; Koster et al., 2014; Patel et al., 2018), requirements concerning content an comprehensibility of the text (Raynor and Bryant, 2013; Pires et al., 2016; Yuan et al., 2019), precision of writing dosing instructions (Borgsteede and Heringa, 2019), and the use of icons, graphics and pictograms (Kheir et al., 2014; van Beusekom et al., 2017). As a result, guidelines have been drawn up with standards on how information should be presented on the PDL (i.e. simple language, one message per PDL line, formulated text as concretely as possible) (Houts et al., 2006; Blake et al., 2010). Also, studies have focused on communication of medicines information, format and organization of the medicines label, as well as number of medicines dispensed (Wolf et al., 2007; Bailey et al., 2012; Emmerton et al., 2012; Samaranayake et al., 2018). There is attention for improving the PDL texts (Maghroudi et al., 2018; Maghroudi et al., 2021), which has improved the labels. However, the ideal approach to bundle these aspects still remains unclear.

Tools have been developed to clarify prescription medication label texts in order to facilitate medication use. For example, medication overviews have been developed using illustrations and icons to support label texts (Dowse et al., 2010; Dowse et al., 2014). These information aids are intended to increase understanding of the usage instructions of prescribed medications (Payne and Avery, 2011; Masnoon et al., 2017), however, there is not yet a good simple solution for patients using multiple medications. A medication overview listing the patient’s medications and use instructions can support patients with polypharmacy to keep a clear overview of their medication use, which in return may lead to better treatment adherence (Nair et al., 2011).

The aim of this study was to understand whether such a personalized medication overview can support patients in their medication use compared to the usual-care PDLs. Our hypotheses were that: 1) patients better understand the medication instructions when they have a personalized medication overview rather than PDLs-only, 2) this understanding increases with the number of medications (the more medications, the greater the benefit from the overview), and 3) a personalized medication overview has influence on the comprehensibility of the medication-use instruction, as it is intended to help patients better process the information on PDL instructions, particularly patients with low health literacy skills.

## Materials and methods

### Design and procedure

#### 2 × 3 between-subjects experimental design

A hypothetical online experiment was set up, comparing groups of respondents exposed vs not exposed to a medication overview and who received PDL instructions for three, five, or eight medications. Participants were divided randomly in six groups (each receiving one of the six questionnaires, for one of the six conditions; n = 150 participants per questionnaire).

#### Participants

Online questionnaires were sent out to panel members of the Nivel Dutch Health Care Consumer Panel, which collects the general population’s experiences and opinions on different matters regarding to healthcare (Brabers et al., 2012). This panel, of approximately 11,500 people (2021) who are 18 years and older from the Netherlands, is an access panel where members have given permission to be contacted to fill in questionnaires on regular basis. The background characteristics of the panel members, such as their gender, age, level, self-reported health status, and education are known. The panel is renewed on a regular basis to ensure that representative samples of the Dutch population can continue to be drawn, with regard to age and gender. Participants are recruited via bought addresses from an address supplier. Panel members are approached about four to five times a year to complete questionnaires, from the approximately eight times to ten per year a survey is distributed on all kinds of topics within the healthcare sector. The respondents are given the choice to fill in a paper or online questionnaire. Respondents can withdraw themselves from the panel at any time, but cannot sign up on own initiative to become member of the panel. Panel members do not receive financial compensation for filling out questionnaires nor is there a membership fee, though by answering the questionnaires they can save up for a gift card.

For the purpose of this study, we approached a sample of 900 members from the Nivel Dutch Health Care Consumer Panel (Brabers et al., 2012). An expert-based opinion was used to determine the appropriate number of respondents, which has also been done in a study with a similar study design (Struikman et al., 2020). First, we selected respondents from previous surveys (2019 and 2020) who indicated they were taking prescription medications, and from the 2019 sample in which health literacy scores were assessed, we also selected respondents who had limited health literacy skills according to their answers/scores on a health literacy scale. This resulted in 811 eligible panel members. Secondly, to complete the total sample of 900, another 89 respondents were sampled at random from the panel. All 900 respondents indicated online as their preference for completing the questionnaire.

#### Stimulus materials

**FIGURE 1:**
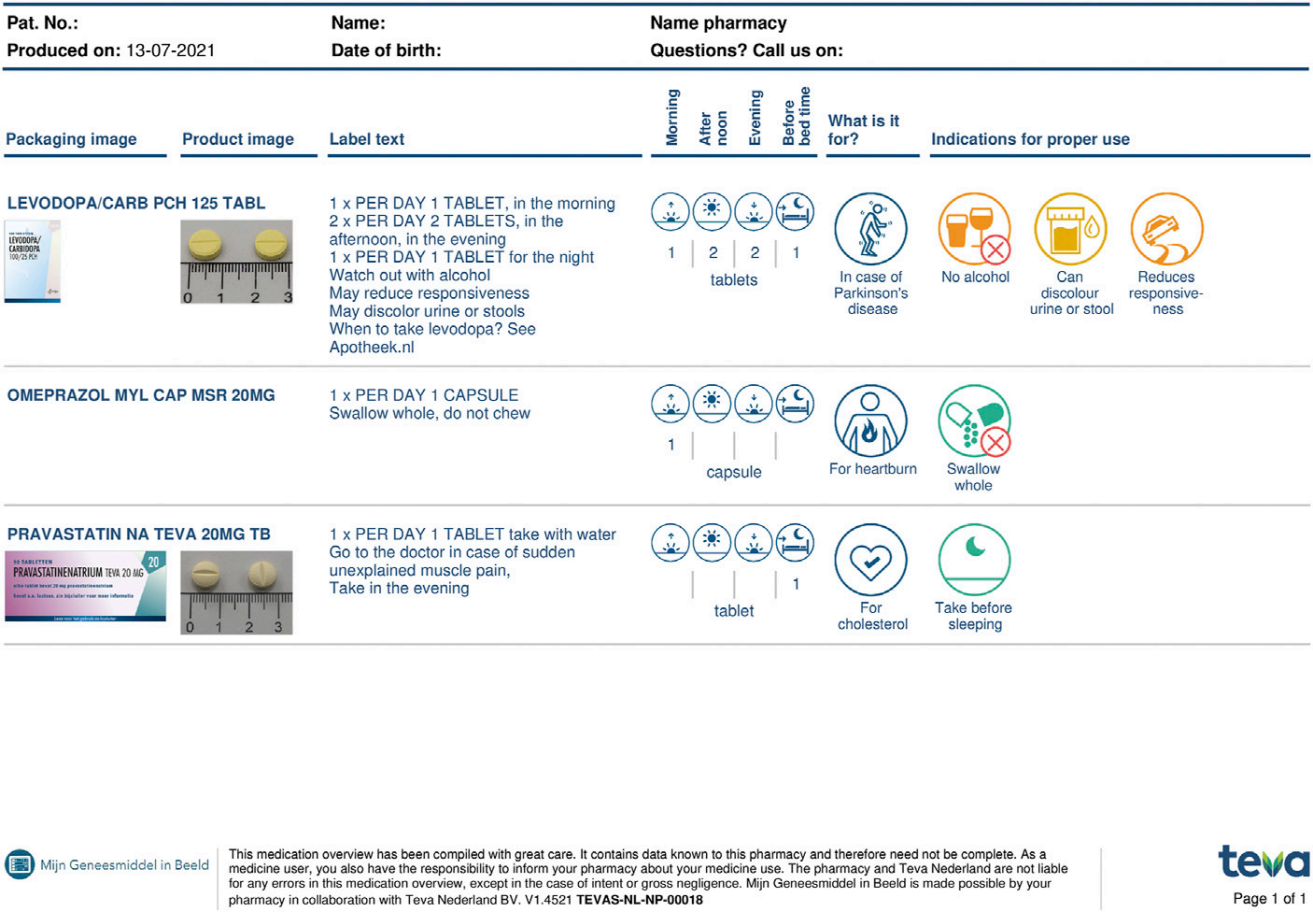
Example of MijnGiB overview for three medications.

**FIGURE 2:**
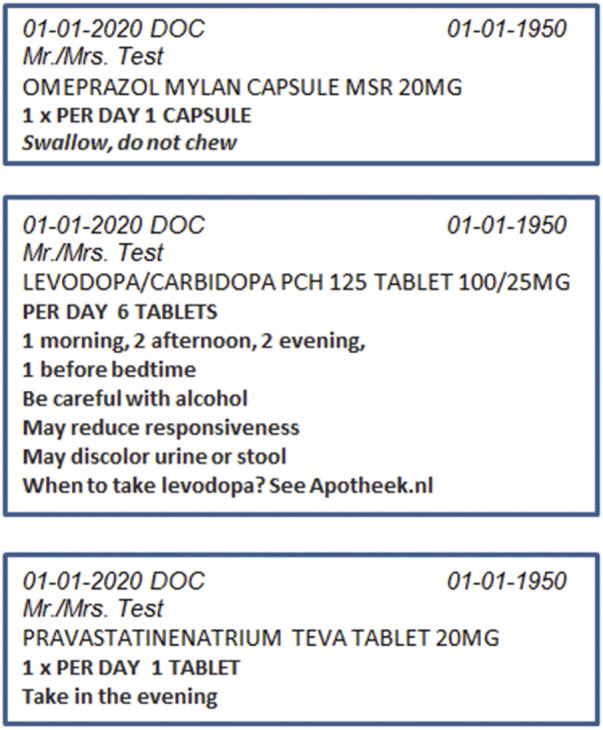
Prescription drug label 1–3 (translated from Dutch to English).

The three ‘exposure’ groups received both the PDLs as used in usual-care as well as the medication overview, My Medication Review (in Dutch: Mijn Geneesmiddel in Beeld^®^ (MijnGiB)) (Figure 1), and the three ‘non-exposure’ groups received PDL instructions only as presented on the medication boxes (Figure 2). Within the conditions, the same medication order was used. The order of the stimulus was also fixed for the participants who received PDL or PDL + MijnGiB.

Since 2019, the pharmaceutical company Teva has been offering MijnGiB, a complete paper version, personalized overview of all medications of the patient in addition to the regular PDL provided by the pharmacy. MijnGiB includes the following information: name of medication, PDL text, moment of intake, the number of tablets per day time, for which condition or disease the medication is used, advice and warnings for use, photos of Teva products to recognize the medication and pictograms/icons of the instructions for proper use.

Both PDLs on medication boxes and MijnGiB communicate dosage instructions and usage advice and warnings. MijnGiB gives more information on the moment of intake, for which condition or disease the medication is taken, as well as photographs of the prescribed medications and tablets/capsules. The additional information on MijnGiB is intended to help patients better process the information on PDL instructions, particularly patients with low health literacy skills.

The respondents were asked to read a hypothetical case (Boxes 1, 2) and to imagine that this hypothetical situation was applicable to them. During the online questionnaire, participants could scroll back to the stimuli material. However, they could not print the stimulus, or at least, this was not presented as an option. The participants were not timed when filling in the questionnaire or viewing the stimulus materials. Questions were asked for a medication with simple (i.e. 1 dose moment per day, 1 tablet) use instructions (omeprazol) and a medicine with more complex instructions (levodopa/carbidopa (L/C)).

BOX 1 **Hypothetical case: situation where three medications are prescribed and MijnGiB and PDLs were provided (translated from Dutch to English) (PDLs and MijnGiB followed after this hypothetical case).**

Imagine being prescribed a new medication by your general practitioner. You go to the pharmacy to pick up this medication. The pharmacy technician (PT) tells you how to take the medication and says that you can also read the instructions on the PDL on the medication box. The PT also gives you two other medications that you have already been using for some time. You can also read on the PDL how to take these medications. In addition, the PT gives you an overview whereby the information is presented in a different way. This overview is called ‘Mijn Geneesmiddel in Beeld’ (MijnGiB). You decide to read this at home. See below the three PDLs and MijnGiB.

BOX 2 **Hypothetical case: situation where three medications are prescribed and PDLs only are provided (translated from Dutch to English) (PDLs followed after this hypothetical case).**

Imagine being prescribed a new medication by your general practitioner. You go to the pharmacy to pick up this medication. The pharmacy technician (PT) tells you how to take the medication and says that you can also read the instructions on the PDL on the package. The PT also gives you two other medications that you have been taking for some time. You can also read on the PDL how to take these medications. You decide to read this at home. See below the three PDLs.

#### Data collection and ethical considerations

The online questionnaire was sent out to the sample of panel members on the 1^st^ of December, 2020, and two reminders were sent on the 8th and 15th of December. The questionnaires closed on the 22nd of December.

According to Dutch legislation, neither obtaining informed consent nor approval by a medical ethics committee is obligatory for carrying out research using the Dutch Healthcare Consumer Panel (CCMO, 2022). Data are analyzed pseudomized, and processed according to the privacy policy of the Dutch Healthcare Consumer Panel, which complies with the General Data Protection Regulation (GDPR) (Supplementary Appendix S1, Data availability). A privacy regulation is accessible for the Nivel Dutch Health Care Consumer Panel (Jong and Brabers, 2022). The research team who analyzed the data had no access to any identifiable information of the respondents, such as name and address. Participation is voluntary and members are not forced to participate in surveys. They can stop their membership at any time without giving a reason.

## Measurements

### Experiment outcome measures

The online questionnaire had four experimental outcome measures (Supplementary Appendix S2, for outcome measure questions from questionnaire with 3 medications, for the MijnGiB + PDLs group). Full questionnaires can be requested from the corresponding author. These were: dosage instructions; 1) how often (x times per day or ‘’I do not know’’), 2) dose timing (morning, afternoon, evening, before bedtime, ‘’I do not know’’), 3) whether it was clear which condition or disease the medication is for (yes/no), and 4) usage advice and warnings. The advice and warnings questions consisted of: which aspects does one need to pay attention to when taking these medications (respondents could select multiple answers, including the options ‘’other’’, or ‘’none of the above’’). We asked the outcome measures for a medication with simple instructions for use (omeprazol) and a medication with more complex instructions (levodopa/carbidopa). At the end we asked if it was clear for which condition or disease the respondent had to hypothetically take the medication (answer options: yes or no).

Given that medication instructions are either followed correctly or incorrectly, we grouped the answers into dichotomous variables. The ‘’I do not know” option was combined with the incorrect answer, with the exception of the question regarding the moment of intake of omeprazol for the condition PDL-only. In practice the PDL text on the medication box corresponds to the PDL text on MijnGiB. In this experiment, the PDL-only did not state at which moment of the day the patient should take their medication. Therefore, the PDL-only group could not have known the answer. Thus, for the respondents who stated “I do not know,’’ this was also classified as a correct answer.

### Background characteristics

Gender, age, education level (low, middle, high) (CBS, 2019), household composition (one person household or multiple person household), ethnicity (native Dutch or (non-) western foreigners), income and perceived general and psychological health on a scale from 1 to 5 (bad, fair, good, very good, excellent) were already known from the panel members. The questions used for the perceived general and psychological health were: In general, how would you describe your general/mental/psychological health? The five-point Likert-scale participants used to answer the questions are based on the categorization of the SF-12 questionnaire (Stewart, 1992).

In addition, questions were asked related to medication use (yes, currently taking one or more prescription medications; no, not at the moment; or no, never used a prescription medication), whether the patient has 1) chronic condition(s) (yes/no), and whether the patient is familiar with MijnGiB (yes (either received from pharmacy or heard of), or no).

### Health literacy score

Chew’s Brief questions to identify patients with inadequate health literacy (SBSQ) tool was used to assess the health literacy of the respondents. Three questions provided insight into their understanding of health information: 1) how often respondents receive assistance in reading health information, 2) their confidence in filling medical forms, and 3) how often the respondents find it difficult to learn more about their health because they do not understand written information. The respondent’s health literacy score was calculated by taking the sum of the three 5-point Likert scale questions, a scale from 0 to 4 (always have problems/not confident to never have problems/confident) (Fransen et al., 2011). An average score of 2 or lower indicates inadequate health literacy, and a score greater than 2 indicates adequate health literacy (Chew, Bradley, Boyko; Chew et al., 2008).

### Statistical analysis

The statistical analysis software STATA version 16 was used to perform the statistical analysis. A p-value of <0.05 was considered statistically significant. Descriptive statistics were used to describe the sample population. A randomization check using one-way ANOVA test (F-test) (Goodall and Appiah, 2008) and chi-square tests (for dichotomous proportions) were performed to examine whether the participant characteristics were equally divided across experimental conditions. One-way ANOVAs were used to analyze differences in proportion of correct answers regarding dosage instructions and advice/warnings between the experiment conditions. Thereby it became it apparent whether there was a statistically significant difference between amount of incorrect and correct answers in the exposed and non-exposed group. The outcome measures were coded dichotomously (0 = incorrect, 1 = correct). Tukey post-hoc tests revealed the difference in means in the groups of respondents with the different experimental conditions. In the case there is a statistically significant difference, the summary of means (SD) gave insight in how much variance there is, e.g. which group (exposed vs non-exposed) had a higher proportion of correct answers than the other group. Two-way full-factorial ANOVA tests were used to analyze interactions.

## Results

Of the 900 invited panel members, 661 responded to the questionnaire and 604 panel members completed the questionnaire fully (response rate 67%). The respondents were almost equally distributed over the six groups, see Table 1. Mean age was 63 years (SD 13). As selected for, most had a chronic condition (79%) and used prescription medications (87%), also almost equally divided over the six groups. The majority had a self-perceived adequate health literacy (96%), implying that the hypothesis on the role of health literacy cannot further be analyzed as the number of respondents with an inadequate health literacy score was too small.

**TABLE 1:** Distribution of participants per condition.

Condition	N (%)
3 medications + MijnGiB	95 (15.7)
5 medications + MijnGiB	101 (16.7)
8 medications + MijnGiB	100 (16.6)
3 medications without MijnGiB	108 (17.9)
5 medications without MijnGiB	98 (16.2)
8 medications without MijnGiB	102 (16.9)

The randomization check presented no significant differences between the six experimental conditions and the participant characteristics. The small group of participants who were familiar with MijnGiB (n = 43) were not equally spread across the six conditions (χ^2^ (5) = 14.4, *p* = 0.01)**.** The participants were therefore excluded from the sample for the data analysis of the experiment, but not for the questions for background characteristics of the sample population. See Table 2 For background characteristics of the respondents.

**TABLE 2:** Background characteristics of respondents (N = 604).

Characteristics	Values	N	Randomization check, p-value
Age (years), mean (SD: range)	62.7 (12.9; 28–90)	604	χ^2^ (5) = 3.8, *p* = 0.6
Gender, n (%)	—	604	χ^2^ (5) = 1.3, *p* = 0.9
Male	305 (50.5)	—	—
Female	299 (49.5)	—	—
Education, n (%)	—	595	χ^2^ (10) = 13.7, *p* = 0.2
Low	56 (9.4)	—	—
Middle	281 (47.2)	—	—
High	258 (43.4)	—	—
Household, n(%)	—	595	χ^2^ (5) = 6.3, *p* = 0.3
One-person household	148 (24.8)	—	—
Multiple-persons household	447 (75.1)	—	—
Migrant status, n(%)	—	597	χ^2^ (5) = 4.5, *p* = 0.5
Non-migrant	546 (91.5)	—	—
Migrant	51 (8.5)	—	—
Health status, n(%)	—	585	χ^2^ (20) = 22.6, *p* = 0.3
Excellent/very good	139 (23.8)	—	—
Good	293 (50.1)	—	—
Fair/bad	153 (26.2)	—	—
Psychological status, n(%)	—	585	χ^2^ (20) = 18.5, *p* = 0.6
Excellent/very good	314 (53.7)	—	—
Good	218 (37.3)	—	—
Fair/bad	53 (9.1)	—	—
Use of prescription medication(s), n(%)	—	604	χ^2^ (1.8) = 1.8 *p* = 0.9
Yes	527 (87.2)	—	—
Has at least one chronic condition, n(%)	—	604	χ^2^ (5.6) = 5.6, *p* = 0.4
Yes	477 (79.0)	—	—
Familiarity with MijnGiB, n(%)	—	599	χ^2^ (14.4) = 14.4, *p* = 0.01
Have heard of MijnGiB	25 (4.2)	—	—
Received MijnGiB from the pharmacy	18 (3.0)	—	—
Never heard or received MijnGiB	556 (92.3)	—	—
Health literacy score, n(%)	—	604	χ^2^ (5) = 7.8, *p* = 0.2
Adequate health literacy _(score >2)_	579 (95.9)	—	—
Inadequate health literacy _(score 2 or lower)_	25 (4.1)	—	—

### 2 × 3 experimental design results

The effect of the instruction type, number of hypothetically prescribed medications, and the interaction effect between the increasing number of medications and instruction type were investigated. No statistically significant differences were found regarding number of medications (three, five, or eight) nor were interaction effects found between the number of medications and instruction type. There were statistically significant differences between the instruction type (non) exposed to the medication overview (Table 3).

**TABLE 3:** Differences in means (SD) between the groups of respondents exposed and non-exposed to MijnGiB (N = 561).

Questions	Non- exposure to MijnGiB (N = 296)	Exposure to MijnGiB (N = 308)	*p*-value
	Correct answers	Correct answers	
	Mean (SD)	Mean (SD)	
levodopa/carbidopa			
How often should you take levodopa/carbidopa?	0.85 (0.4)	0.76 (0.4)	0.02
At what moment of the day should you take levodopa/carbidopa?	0.92 (0.3)	0.86 (0.4)	0.04
Is it clear for which condition, disease or ailment you should use levodopa/carbidopa?	0.03 (0.2)	0.83 (0.4)	<0.001
Which of the following should you watch out for while taking levodopa/carbidopa?	0.91 (0.3)	0.89 (0.3)	0.5
omeprazol			
How often should you take omeprazol?	0.96 (0.2)	0.96 (0.2)	0.9
At what moment of day should you take omeprazol ?	0.80 (0.4)	0.80 (0.4)	1.0
Is it clear for which condition, disease or ailment you should use omeprazol ?	0.10 (0.3)	0.85 (0.4)	<0.001
Which of the following should you watch out for while taking omeprazol ?	0.93 (0.3)	0.93 (0.3)	0.8
Medications received			
If you look at all PDLs, for which conditions, diseases or ailments have you have received medications?	0.04 (0.2)	0.66 (0.5)	<0.001

### Dosage instructions

#### How often one takes medication

In total, there was a high proportion of correct answers (mean 0.81, SD 0.4) for how often one should take L/C per day in the exposed (E) and non-exposed (NE) groups to MijnGiB (n = 541). There was a significant difference in the proportion of correct answers amongst the two groups. The non-exposed group gave a slightly higher proportion of correct answers for how often (mean 0.85, SD 0.4 NE; mean 0.76, SD 0.4 E, *p* = 0.02) one should take L/C per day. There were no significant differences for how often one should take omeprazol. In both groups of the respondents (n = 535), there was the same proportion of respondents who gave the correct answer (mean 0.96, SD 0.2) for the non-exposed and exposed group to MijnGiB.

#### Moment of intake per day

There was also a high proportion (mean 0.89, SD 0.3) of the total respondents (n = 542) who gave the correct answer on the question about at which moment of the day one should take L/C. The non-exposed group had a slightly higher proportion of correct answers (mean 0.92, SD 0.3 NE; mean 0.86 SD, E, *p* = 0.04). Of the total group respondents (n = 533) who answered the question on which moment of the day they should take omeprazol, there was an overall high proportion of correct answers given (mean 0.8, SD 0.4). This correct answer includes respondents in the PDL-only group who stated ‘’I do not know’’ given that the information was not present on the PDL. There was no significant difference in the proportion of the correct answers between the two groups (mean 0.80, SD 0.4 E; mean 0.80, SD 0.4 NE, *p* = 1.0).

#### Medication use for type of condition or disease

In total, respondents (n = 539) gave a lower proportion of correct answers (mean 0.40, SD 0.5) for which condition or disease the medication is used. There was a significant difference in the proportion of correct answers between the two groups. The exposed respondents gave a higher proportion of right answers for which condition or disease they should use L/C (mean 0.83, SD 0.4 E; mean 0.03, SD 0.2 NE, *p* < 0.001). In comparison, the respondents (n = 540) also gave a lower proportion of right answers (mean 0.45, SD 0.5) regarding for which condition or disease omeprazol is used. There was a significant difference between the groups. MijnGiB-exposed respondents gave a higher proportion of correct answers for which condition or disease they should use omeprazol (mean 0.85, SD 0.4 E; 0.10, SD 0.3 NE, *p* < 0.001).

#### Medication usage advice and warnings

Overall, there was a high proportion (mean 0.9, SD 0.3) of correct answers amongst the respondents (n = 409) who answered the question on what they should pay attention to when using L/C. Also, for omeprazol, of the total respondents (n = 496) a high proportion gave the correct answer (mean 0.93, SD 0.3). No significant differences in the proportion of the correct answers between the exposed and non-exposed group were found.

#### Overview of medications respondents received

At the end of the experiment questions, respondents were asked for which conditions, diseases or ailments they had received the instruction labels. A small proportion (mean 0.3, SD 0.5) of the total respondents (n = 545) gave the right answer. There was a significant difference in the proportion of the correct answers between the exposed and non-exposed group. MijnGiB-exposed (E) respondents gave a higher proportion of correct answers for the questions regarding for which medications they received the instruction labels compared to the non-exposed group (mean 0.66, SD 0.5 E; mean 0.04, SD 0.2 NE, *p* < 0.001).

## Discussion

In this study, we reported on the added value of a personalized medication overview to support patients in their medication use compared to usual-care PDLs. The majority of the respondents gave a high proportion of correct answers, despite the type of PDL instruction, indicating high comprehensibility of both the usual-care PDL instructions and on the personalized medication overview. Respondents exposed to the medication overview gave a higher proportion of correct answers compared to non-exposed for instructions on usage advice (additional information presented on the medication overview) for both a medication simple and complex use instructions. Regarding dose timing (how much and at what moment) of the simple medication both groups gave the same proportion of correct answers. A greater proportion of respondents exposed to the usual-care PDL only gave correct answers for how often and dose timing of the more complex medication. No differences were found regarding number of medications nor were interaction effects found between the number of medications and information type. The results show that a medication overview can be a good addition (as it contains additional information) to support patients in their medication use compared to usual-care PDLs.

Problems understanding medical information seem to be more common in certain patient groups, such as the elderly, people with limited health literacy, and people with language barriers (van Dijk et al., 2016). In this study, not all these factors were investigated. We had a selective population with older medication users with adequate health literacy skills, making it not comparable to the literature that up to 50% of the adult population incorrectly understands the dosage information on PDLs (Davis et al., 2006a; Davis et al., 2006b; Wolf et al., 2006; Wolf et al., 2007; Bailey et al., 2009; Wolf et al., 2010; Wolf et al., 2011; Bailey et al., 2012; Bailey et al., 2014).

The medication overview had beneficial effects on understanding for which condition or disease the medication should be used. This turned out to be the case regardless of whether it was a medication with simple or more complex instructions for use, and regardless of the number of other medications someone is taking according to the hypothetical scenario. It is thereby important to mention that on the PDL, there is only limited space, making it difficult to provide additional information (Maghroudi et al., 2020).

Including specifically the medicine use information (intake, dosing moment) on the PDL is important for patient safety. An additional overview, such as the MijnGiB, is a good way to provide more information that does not fit on the prescription medication label.

The effects of the medication overview on understanding how to take the medication depended on whether it was a medication with simpler or more complex instructions for use. For the medication with simpler instructions for use (omeprazol), the addition of the medication overview had no effect for understanding how the medication should be taken. For the medication with more complex instructions for use (L/C), the addition of the medication overview had less of an effect than the PDL-only, as the group respondents with the usual-care PDL-only had higher percentages of correct answers. It might be plausible that the participant has an information preference and chooses one information type over the other. Hence, in the situation that the participant received both types of information, it could have been possible they choose the PDL over the medication overview.

There are different reasons that could explain why respondents with the usual-care PDL-only had a higher proportion of correct answers. For example, there is less information on the usual-care PDL, and thus less information to understand, whereby the core information is highlighted more easily. Respondents may also be used to using the usual-care PDLs as the majority of the respondents use medications in their own day-to-day lives. Thus, the usual-care PDLs may have been easier to use during the experiment than a medication overview like MijnGiB as it is new. This latter may be specifically applicable to the older respondents, who were overrepresented in this study due to our sample stratification. Research conducted on how elderly think about change indicates that they often want things to stay the way they are (Molenaar, 2022). Therefore, as long as an older person can still get away with their way of doing things, like the use of the usual-care PDLs, they will probably opt for this rather than a new development like a personalized medication overview.

Moreover, we hypothesized that patients better understand the medication instructions when they have a personalized medication overview rather than PDLs-only, and that this understanding increases with the number of medications. However, there were no significant differences found regarding number of medications, nor were interaction effects found between the number of medications and instruction type. A possible explanation might be linked to the setup of the experiment as all respondents were asked how well they understand the instructions for use (at what moment and how often) of one specific medication at a time and not all three, five or eight. The results show this is slightly easier to do with the PDL-only of this specific medication than when the personalized medication overview is added. This may be the case because the personalized medication overview provides information about the use of several medications at the same time, which may suggest that use of specifically one of these medications (omeprazol or L/C) becomes more omitted. When measuring how well people understand the use of one medication at a time, the medication overview may be less beneficial as opposed to only the PDL with one medication.

### Strengths and limitations

A strength of this study is that we used a controlled experimental design, in which the respondents were randomly assigned across the six conditions, and the groups were equally spread with regard to the background characteristics (i.e. age, education level, *etc.*) of the respondents. Another strength is the use of the Dutch Health Care Consumer Panel, which includes people who cannot register themselves, but have to be invited to join the panel. In panels that are formed by people signing up on their own to join, there is higher risk of selection bias. Our panel includes people who do accept an invitation, but would not register themselves.

There were also limitations to this study. A limitation of this study can be the hypothetical situation of this experiment. Respondents were asked to imagine a situation in which they are prescribed several medications. This might have been difficult for some respondents, especially since most of them already use medication in their own daily life, and answered the questions based on their own experiences. They might have responded differently if it was their own medication they were asked about. Nevertheless, as shown in the meta-analysis by Van Vliet et al. (Van Vliet et al., 2012), results of actual patients would not have been stronger than using analog patients/fictive examples, as in this study.

Another limitation of this study is that there was little or no variation in the health literacy (on the health literacy scale used for this study) of the respondents in this sample. This sample was selected for limited health literacy, yet the vast majority self-identified adequate health literacy. The small group of people with inadequate health literacy may be related to ease or difficulty that people with a lower health literacy may experience when completing questionnaires.

In addition, a limitation is that respondents were not given an instruction on how to use the medication overview. In the pharmacy one does receive an explanation on how to use the medication overview, which might make it easier to use the overview, and prevent potential misunderstandings of medicine use information. Furthermore, a limitation is that it was not known whether people in the experiment sample took the specified medications as we present in the experiment.

Lastly, a limitation of this study could be reflected on the setup of the experiment and the outcomes on how well the participants understood the medicine use information for three, five, or eight medicines. All respondents were asked how well they understood the instructions for use (when and how often) of one specific medication at a time. Measuring how well people understand the use of one medication at a time, MijnGiB may be less beneficial as opposed to the prescription medication label only with one medication. However, more positive effects from MijnGiB may be expected from how well people understand the use of all medications together when comparing MijnGiB and the prescription medication label only.

### Implications for research

The results of this study do not fully assess how the medication overview may help people with low literacy given the small group of respondents (4%) with low literacy. Future research can focus on better identifying patient groups for whom the personalized medication offers the most support. Also, the medication overview appears to be less beneficial when measuring how well people understand the use of one of the medications. However, more positive effects can be expected from how well people understand the use of all medications together. The latter has not been measured, but is a suggestion for further research. For further research it is also important to test in real life conditions. For example, with patients using their own personalized medication overview, how do they understand the usage information and what are their impressions for their own medication use. Moreover, this study focused on oral medicines (tablets), and could be extended to dosage forms with more complex instructions (e.g. variable dosing) or mastery of technique for self-administration of the medicine in future studies.

## Conclusion

Both the respondents who were shown the personalized medication overview and the respondents who only saw the PDLs showed a high level of comprehensibility of the use instructions for the hypothetically prescribed medications. However, the medication overview increased respondents’ comprehension of the instructions regarding the usage advice and for which condition or disease one should use the medication, which is extra information on this overview. The overview can be a good addition to the prescription drug labels to support patients in their medication use. Future research should focus on identifying patient groups who might benefit more, by testing the use of a medication overview among different patients.

## Data Availability

The raw data supporting the conclusions of this article will be made available by the authors, without undue reservation.
