# Supplementary material for: Comprehensibility of a personalized medication overview compared to usual-care prescription drug labels
Source: Front Pharmacol. 2022 Oct 28;13:1004830. doi: 10.3389/fphar.2022.1004830 (PMC9650257; doi:10.3389/fphar.2022.1004830)
Supplement: Supplementary file 1 [file DataSheet2.docx]

**S2 Appendix 2, outcome measure questions from questionnaire with 3 medications, MijnGiB + PDLs**

You have just read the prescription drug labels and MijnGiB. We would now like to ask you to answer the following questions for the medication levodopa/carbidopa. So it is not about your own medication use!

1. How often should you take levodopa/carbidopa?

... once a day

- I don't know

2. At what time of day should you take levodopa/carbidopa? (Multiple answers possible)

- Morning
- Noon
- Evening
- Before bed
- I don't know

3. Is it clear for which condition, disease or ailment you should use levodopa/carbidopa?

- Yes, namely for ....
- No, that is not clear

4. Which of the following aspects should you pay attention to when using levodopa/carbidopa? (Multiple answers possible)

- I have to swallow the tablet in 1x completely (so do not chew)
- I need to dissolve the tablet in water
- I have to take the medication with plenty of water
- I should not drink grapefruit juice now that I am taking this medication
- I have to be careful when driving
- I should not drink alcohol now that I am taking this medication
- I need to take the medication with meals
- I need to take the medication after meals
- I need to take the medication before meals
- My response may be delayed
- I need to keep the medication in the refrigerator
- I need to take all the tablets prescribed
- The drug has a limited shelf life
- Otherwise, namely ...............................................
- None of the above aspects

We would now like to ask you to answer the same questions for another medication, namely omeprazol These questions are therefore not about your own medicines, if any.

5. How often should you take omeprazol?

... once a day

- I don't know

6. At what time of day should you take omeprazol ? (Multiple answers possible)

- Morning
- Noon
- Evening
- Before bed
- I don't know

7. Is it clear for which condition, disease or ailment you should use omeprazol?

- Yes, namely for ....
- No, that is not clear

8. Which of the following things should you pay attention to when using omeprazol? (Multiple answers possible)

- I have to swallow the tablet in 1x completely (so do not chew)
- I need to dissolve the tablet in water
- I have to take the medication with plenty of water
- I should not drink grapefruit juice now that I am taking this medication
- I have to be careful when driving
- I should not drink alcohol now that I am taking this medication
- I need to take the medication with meals
- I need to take the medication after meals
- I need to take the medication before meals
- My response may be delayed
- I need to keep the medication in the refrigerator
- I need to take all the tablets prescribed
- The drug has a limited shelf life
- Otherwise, namely ...............................................
- None of the above aspects

9. If you look at all three labels and MijnGiB, for which conditions, diseases or ailments have you received medications? (mark up to three answers)

- High blood pressure
- Increased cholesterol
- Diabetes
- Astma / COPD
- Rheumatism or joint complaints
- Osteoporosis
- Stomach- of intestine problems
- Depression
- Parkinson's disease
- Pain
- Allergies
- Skin complaints
- Otherwise, namely .......
- I don't know
