# Supplementary material for: Comprehensibility of a personalized medication overview compared to usual-care prescription drug labels
Source: Front Pharmacol. 2022 Oct 28;13:1004830. doi: 10.3389/fphar.2022.1004830 (PMC9650257; doi:10.3389/fphar.2022.1004830)
Supplement: Supplementary file 2 [file DataSheet1.docx]

# **S1 Appendix 1. Data availability**

The Dutch Health Care Consumer Panel has a program committee, which supervises processing the data of the Dutch Health Care Consumer Panel and decides about the use of the data. This program committee consists of representatives of the Dutch Ministry of Health, Welfare and Sport, the Health Care Inspectorate, Zorgverzekeraars Nederland (Association of Health Care Insurers in the Netherlands), the National Health Care Institute, the Federation of Patients and Consumer Organizations in the Netherlands, the Dutch Healthcare Authority and the Dutch Consumers Association. All research conducted within the Consumer Panel has to be approved by this program committee. The committee assesses whether a specific research fits within the aim of the Consumer Panel, that is strengthen the position of the health care user. Data are available upon request from Judith D. de Jong, PhD ([j.dejong@nivel.nl](mailto:j.dejong@nivel.nl)), project leader of the Dutch Health Care Consumer Panel.
